# Characterization of *Bacillus stercoris* JK-6 as an Antifungal Agent Against Crop Fungal Diseases

**DOI:** 10.3390/jof12070467

**Published:** 2026-06-25

**Authors:** Qing Ouyang, Jiazheng Wang, Xiangyan Liu, Siyang Wang, Zirui Chen, Huabin Zhou, Xiaolin Chen, Xiang Lu, Qing Xiong, Jia Su, Tuo Qi, Xuewei Chen, Min He

**Affiliations:** 1 State Key Laboratory of Crop Gene Exploration and Utilization in Southwest China, Sichuan Agricultural University, Chengdu 611130, China; qingouyang6269@163.com (Q.O.); wangjiazheng0918@163.com (J.W.); lxy2429919344@163.com (X.L.); wsy305026341@163.com (S.W.); zrchen1020@163.com (Z.C.); 15282627576@163.com (H.Z.); chenxl1112025@163.com (X.C.); luxiang@sicau.edu.cn (X.L.); xiongqing_cyan@163.com (Q.X.); 15228131537@163.com (J.S.); 2 Ecological Security and Protection Key Laboratory of Sichuan Province, Mianyang Teachers’ College, Mianyang 621000, China; qituo777@163.com

**Keywords:** *Bacillus stercoris*, biological control, crop, fungal disease, surfactin

## Abstract

Biological control is one of the most effective strategies for managing crop fungal diseases such as rice blast, which severely threatens global food security. However, the limited availability of microbial biocontrol resources and incomplete understanding of their mechanisms hinder the development of practical biocontrol technologies for rice blast. In this study, a *Bacillus stercoris* strain, JK-6, isolated from the rhizosphere soil of rice, was identified as a promising biocontrol agent with strong antagonistic activity against multiple fungal pathogens. The fermentation broth of JK-6 yielded inhibition rates of 94.96% against *Magnaporthe oryzae* (rice blast), 75.83% against *Bipolaris maydis* (maize southern leaf blight), and 70.46% against *Fusarium graminearum* (wheat head blight). Whole-genome sequencing of JK-6 revealed 12 biosynthetic gene clusters, one of which was responsible for the biosynthesis of the lipopeptide surfactin. Further assays showed that 200 μM surfactin exhibited broad-spectrum antifungal activity, with inhibition rates of 82.90%, 66.76%, and 52.54% against *M. oryzae*, *B. maydis*, and *F. graminearum*, respectively. Mechanistically, surfactin suppresses fungal growth by downregulating genes involved in integral and intrinsic membrane components and oxygen transport, as validated by transcriptomic analysis. Our discoveries not only advance the conceptual understanding of the surfactin-mediated JK-6 antagonistic activity against fungal diseases but also offer an effective new approach for the practical control of crop fungal diseases.

## 1. Introduction

Global agricultural productivity and crop quality are significantly threatened by the prevalence of fungal diseases [1,2]. The production and quality of rice, one of the world’s major food crops, face severe challenges from rice blast disease, caused by the fungal agent *Magnaporthe oryzae* [3]. Rice blast can lead to annual yield losses of 10–30% and, in severe cases, complete crop failure. Therefore, mitigating the impact of rice blast is crucial for ensuring safe rice production. Currently, several chemical fungicides are commercially registered for rice blast management. However, issues such as resistance development and harmful ecological effects restrict their long-term sustainable application, driving the urgent exploration of alternative biocontrol resources [4]. Biological control, in which metabolites from microbes or plants are used as bioactive fungicides to inhibit pathogen growth and infection, represents one of the most sustainable and environmentally friendly disease management strategies [5]. Identifying microorganisms with potent, broad-spectrum biocontrol activity and characterizing their active fungicidal compounds are fundamental to the development of biological control measures.

Beneficial microorganisms have been used for biocontrol of rice blast since the 1950s, particularly genera such as *Bacillus*, *Streptomyces*, *Pseudomonas*, and *Trichoderma* [6]. For example, *Streptomyces* sp. PM5 exhibits strong antagonistic activity against rice blast through the antifungal compound SPM5C-1 [7]. A biocontrol strain of *Bacillus amyloliquefaciens*, LM-1, significantly inhibits the germination of blast fungus spores and cell wall formation [8]. Furthermore, *Pseudomonas mosselii* strain 923 is highly effective in controlling various diseases via its natural antibiotic pseudoiodinine [9]. These bioactive fungicides impede fungal diseases through various mechanisms, including the inhibition of pathogenic microbial growth and reproduction, modification of host plant resistance, and activation of the plant’s defense responses. However, due to insufficient identification of active molecules and a lack of in-depth understanding of biocontrol mechanisms, resources that can be effectively applied to manage various fungal diseases remain limited.

*Bacillus stercoris* was classified as a new *Bacillus* species based on chemotaxonomy and phenotyping in 2020 [10]. Similarly to other *Bacillus* species, *B. stercoris* can secrete antimicrobial metabolites to inhibit biofilm formation by pathogenic bacteria. Previous studies of *B. stercoris* have covered diverse research fields, including plant and animal disease biocontrol, environmental protection, and aquaculture disease resistance, but few reports focus on its antifungal metabolites against phytopathogens. The *B. stercoris* strain B.PNR1 was found to exhibit significant phosphate solubilization ability, cellulase activity, and inhibitory effects against the tomato wilt pathogen while promoting plant growth [11]. In a separate study, the *B. stercoris* strain DXQ-1 isolated from soil samples exhibited robust antagonistic effects against the rice blast pathogen *M. oryzae* by disrupting fungal hyphal integrity and suppressing conidial germination, with its crude extract achieving a 65.86% inhibition rate against *M. oryzae* [12]. Additionally, the *B. stercoris* strain 92p was found to secrete antifungal metabolites that effectively combat *Neocordana musae*, offering a sustainable approach to managing diseases in banana production [13]. Although previous studies have isolated numerous *B. subtilis* strains with significant biocontrol potential, the bioactive metabolites produced by these strains remain inadequately identified, thus hampering the advancement of novel biofungicides based on this species.

Surfactin, a naturally occurring cyclic lipopeptide, has garnered considerable attention in the field of plant protection due to its broad range of biological activities. This biosurfactant is predominantly produced by bacteria of the genus *Bacillus* [14,15]. Surfactin exhibits potent, broad-spectrum antibacterial, antiviral, and antifungal properties, rendering it a promising candidate for the biological control of plant diseases. Because of its lipophilic nature, surfactin can compromise the structural integrity and physiological function of cell membranes, leading to the lysis of targeted pathogenic microorganisms [16]. Surfactin also enhances rhizosphere colonization and biofilm formation by beneficial microbial strains to help maintain a favorable rhizosphere environment [17]. Given its environmentally benign properties and multifunctional benefits, surfactin serves as a sustainable alternative to synthetic biopesticides and holds significant promise for application in integrated pest management programs. However, it remains largely unknown how surfactin inhibits the growth- or development-associated molecular processes of fungal pathogens, thus restricting its large-scale practical application [18,19].

The present study was designed to characterize the antifungal potential of *B. stercoris* JK-6 isolated from rice rhizosphere soil against three important crop pathogenic fungi (*M. oryzae*, *F. graminearum*, and *B. maydis*). Whole-genome sequencing was performed to screen gene clusters involved in secondary metabolite biosynthesis and predict bioactive lipopeptide synthesis genes. Combined untargeted metabolomics and HPLC–MS/MS were further used to confirm the production of surfactin by JK-6, while transcriptomic analysis was conducted to explore the molecular mechanism underlying surfactin’s suppression of *M. oryzae* growth.

## 2. Materials and Methods

### 2.1. Isolation and Identification of JK-6

The biocontrol bacterium was isolated from the rhizosphere soil of rice using a co-culture method against *M. oryzae* in CM, as previously described [20]. The soil samples were obtained from a rice cultivation area in the Wenjiang District of Chengdu, Sichuan Province, China. The bacterial strain JK-6, demonstrating significant inhibitory effects on the growth of *M. oryzae*, was selected for subsequent analyses. The DNA fragment coding for 16S ribosomal RNA was amplified using specific primers (27F: AGAGTTTGATCMTGGCTCAG; 1492R: CGGTTACCTGTTACGACTT), and phylogenetic analysis was conducted by the neighbor-joining (NJ) method using the MEGA 7.0 software (RRID: SCR_011920) [21].

### 2.2. Genome Sequencing, Assembly, and Annotation of JK-6

The complete and accurate genome sequences of JK-6 were determined using a standard protocol combing second- and third-generation sequencing technologies. High-quality genomic DNA was extracted from *B. stercoris* JK-6 using the STE (Sucrose–Tris–EDTA) method [22]. The harvested DNA was detected via agarose gel electrophoresis and quantified using a Qubit^®^ 2.0 Fluorometer to ensure integrity and quality (Thermo Fisher Scientific, Waltham, MA, USA), and then used for sequencing through the Nanopore PromethION and Illumina NovaSeq PE150 platforms (Beijing Novogene Bioinformatics Technology Co., Ltd., Beijing, China). For the second-generation sequencing technology, sequencing libraries were constructed with the NEBNext^®^ UltraTM DNA Library Prep Kit for Illumina (New England Biolabs, Ipswich, MA, USA). For the third-generation sequencing technology, qualified genomic DNA was fragmented using G-tubes (Covaris, Woburn, MA, USA) and then end-repaired to prepare libraries for Nanopore sequencing (with a fragment size > 10 kb using the Blue Pippin system), according to the manufacturer’s instructions (Pacific Biosciences, Menlo Park, CA, USA). Unicycler (https://github.com/rrwick/Unicycler, version: 0.4.8, accessed on 29 August 2023) [23] was used to combine the PE150 and Nanopore data to assemble reads, which were then compared to the assembled sequence, and the distribution of sequencing depth was counted.

We used six databases to predict gene functions: GO (Gene Ontology), KEGG (Kyoto Encyclopedia of Genes and Genomes), COG (Clusters of Orthologous Groups), NR (Non-Redundant Protein Database), TCDB (Transporter Classification Database), and Swiss-Prot. A whole-genome Blast search of JK-6 (E-value less than 1 × 10^−5^; minimal alignment length percentage larger than 40%) was performed against the above six databases. The secretory proteins were predicted using the SignalP database (Version 4.1) [24]. Meanwhile, the secondary metabolism gene clusters we analyzed using antiSMASH 8.0 [25].

### 2.3. Bacterial Strains and Growth Conditions

JK-6 was cultured on potato dextrose agar (PDA) medium at 28 °C. One liter of PDA medium contained 7 g of potato infusion powder, 20 g of dextrose, and 15 g of agar, and the final volume was adjusted to 1 L with distilled water. The blast fungus *M. oryzae* strain Guy11 was initially cultured on complete medium (CM) at 25 °C for 8 days and then transferred to tomato oat medium for sporulation at 25 °C for 2 days [26]. After the mycelia were fragmented, the culture was incubated at 25 °C for an additional 2 days. Plant pathogenic fungi, including *F. graminearum* [27], *R. solani* [28], and *B. maydis* [29], were cultured and stored following standard methods.

### 2.4. Crude Extraction of JK-6 Fermentation Broth

Strain JK-6 was cultivated in potato dextrose liquid medium at 28 °C on a rotary shaker for 48 h. The fermentation broth was then collected, mixed with an equal volume of ethyl acetate (1:1, *v*/*v*), and shaken vigorously to ensure complete extraction of metabolites into either aqueous or organic phase. The aqueous and organic phases were separated, and the organic layers were collected. The solvent was removed from the combined organic extracts using a rotary evaporator at 40 °C, yielding a viscous brown residue. This residue was dissolved in HPLC-grade methanol to a final concentration of 1 mg/mL, and the resulting solution was used for non-targeted metabolomics analysis.

### 2.5. Test of Surfactin’s Antagonistic Effect on M. oryzae Growth

A stock solution of surfactin standard (Shanghai Yuanye Bio-Technology Co., Ltd., Shanghai, China) was prepared using ddH_2_O and sterilized by filtration through a 0.22 μm water phase filter for later use. An appropriate amount of the surfactin stock solution was added to the CM to achieve final concentrations of 50 μM, 100 μM, 200 μM, 300 μM, and 400 μM surfactin. Next, 7 mm diameter agar blocks of *M. oryzae* were inoculated onto the center of the prepared CM plates with different concentrations of surfactin, as well as onto CM plates without surfactin, which served as the control. The plates were incubated at 25 °C for one week, and then the colony diameters were measured. The inhibition of *M. oryzae* growth was quantified using the following formula: inhibition rate (%) = [((dc − 7) − (dt − 7))/(dc − 7)] × 100, where dc and dt represent the colony diameters in the control and treatment groups, respectively.

### 2.6. Untargeted Metabolomics Analysis

To identify specialized metabolites produced by strain JK-6, the ethyl acetate extract from the fermentation broth of JK-6 was analyzed via UPLC–MS/MS (Bioprofile Biotechnology Co., Ltd., Shanghai, China). Concurrently, uninoculated PDB liquid medium was set as the control and subjected to identical extraction procedures. Briefly, 100 µL of sample was thoroughly mixed with 400 µL of cold methanol acetonitrile (1:1, *v*/*v*) by vortexing. The mixture was then sonicated in an ice bath for 1 h, incubated at −20 °C for 1 h, and centrifuged at 14,000× *g* for 20 min at 4 °C. The supernatant was collected and dried under vacuum for LC-MS analysis.

Metabolomics profiling was performed using a UPLC-ESI-Q-Orbitrap-MS system consisting of a Shimadzu Nexera X2 LC-30AD UHPLC (Shimadzu Corporation, Kyoto, Japan) coupled with Q-Exactive Plus (Thermo Fisher Scientific, San Jose, CA, USA). Liquid chromatography (LC) separation was carried out on an ACQUITY UPLC^®^ HSS T3 column (2.1 × 100 mm, 1.8 μm; Waters, Milford, MA, USA). The mobile phase consisted of 0.1% formic acid in water (A) and 100% acetonitrile (B) at a flow rate of 0.3 mL/min. The gradient program was 0% buffer B for 2 min and was linearly increased to 48% in 4 min, then up to 100% in 4 min and maintained for 2 min, and then decreased to 0% buffer B in 0.1 min, with a 3 min re-equilibration period; the column temperature was 40 °C. The HESI source conditions were set as follows: Spray Voltage: 3.8 kv (positive) and 3.2 kv (negative); Capillary Temperature: 320 °C; Sheath Gas (nitrogen) flow: 30 arb (arbitrary units); Aux Gas flow: 5 arb; and Probe Heater Temp: 350 °C.

Raw LC–MS/MS data were processed via MS-DIAL for peak alignment, retention time calibration, and peak area extraction. Metabolite identification relied on accurate precursor mass (<10 ppm) and fragment MS/MS tolerance (<0.02 Da), with spectrum matching against HMDB, massbank, and other public databases. In the extracted-ion features, only variables with more than 50% of nonzero measurement values in at least one group were kept. Multivariate statistical analyses were performed in R v4.0.3 after Pareto scaling of normalized data, including PCA, PLS-DA, and OPLS-DA. Model overfitting was tested via 200-permutation tests, assessed by cumulative R2X, R2Y, and Q2. Candidate differential metabolites were screened with dual thresholds, VIP > 1.0 from OPLS-DA and *p* < 0.05 from one-way ANOVA, accompanied by fold-change calculation and subsequent clustering analysis. All LC-MS/MS experiments were conducted in biological triplicates, and the data have been deposited in the OMIX of the China National Center for Bioinformation (https://ngdc.cncb.ac.cn, accessed on 8 June 2026) under accession code PRJCA066201.

### 2.7. RNA Extraction and Transcriptomic Analysis

Total mycelial RNA was extracted from *M. oryzae* in the surfactin treatment group and in the control group using the TRIzol Reagent (Invitrogen, Thermo Fisher Scientific, Carlsbad, CA, USA). Approximately 3 μg of RNA was used as input material for the RNA sample preparations. Sequencing libraries were generated according to the following steps. Firstly, mRNA was purified from total RNA using poly-T oligo-attached magnetic beads. Fragmentation was carried out using divalent cations at an elevated temperature in an Illumina proprietary fragmentation buffer. First-strand cDNA was synthesized using random oligonucleotides and SuperScript II. Second-strand cDNA synthesis was subsequently performed using DNA Polymerase I and RNase H. Remaining overhangs were converted into blunt ends via exonuclease/polymerase activities, and the enzymes were removed. After adenylation of the 3′ ends of the DNA fragments, Illumina PE adapter oligonucleotides were ligated to prepare them for hybridization. The library fragments were purified using the AMPure XP system (Beckman Coulter, Beverly, CA, USA), and cDNA fragments with the preferred 400–500 bp length were selected. DNA fragments with ligated adaptor molecules on both ends were selectively enriched using Illumina PCR Primer Cocktail in a 15-cycle PCR reaction. Products were purified (AMPure XP system) and quantified using the Agilent high-sensitivity DNA assay on a Bioanalyzer 2100 system (Agilent, Santa Clara, CA, USA).

The sequencing library was sequenced on a NovaSeq X plus platform (Illumina). High-quality sequences (Clean Data) were processed using the Cutadapt (v1.15) software from raw data for further analysis. We aligned reads of all samples to the *M. oryzae* reference genome using HISAT2 v2.0.5. Differential gene expression analysis was performed using DESeq (1.30.0), with screening conditions as follows: expression difference multiple |log_2_FoldChange| > 1, and significant *p*-value < 0.05. Differentially expressed genes were then subjected to GO function and KEGG pathway enrichment analyses. The data have been deposited in the GSA of the China National Center for Bioinformation (https://ngdc.cncb.ac.cn, accessed on 17 June 2026) under accession code PRJCA050265.

### 2.8. qRT-qPCR Assay

Twelve differentially expressed genes (DEGs), six upregulated and six downregulated, were selected for quantitative real-time PCR (qRT-PCR) analysis to validate the reliability of the RNA-seq data. Total RNA was extracted from the same batch of mycelial samples used for transcriptome sequencing, following the method described above. cDNA was synthesized using a reverse transcription kit (Vazyme, Nanjing, China). qRT-PCR was performed on a Bio-Rad CFX96 Real-Time System (Bio-Rad, Hercules, CA, USA). *M. oryzae* β-Tubulin (MGG_00604) was used as an internal reference for normalization. Each RT-qPCR assay was repeated with at least two more biological replicates independently with three technical replicates per sample. The 2^−∆∆CT^ method was used to calculate the relative expression levels with three technical replicates. All primer sequences used in this study are listed in Table S2.

## 3. Results

### 3.1. Identification of Biocontrol Strain JK-6

**Figure 1:**
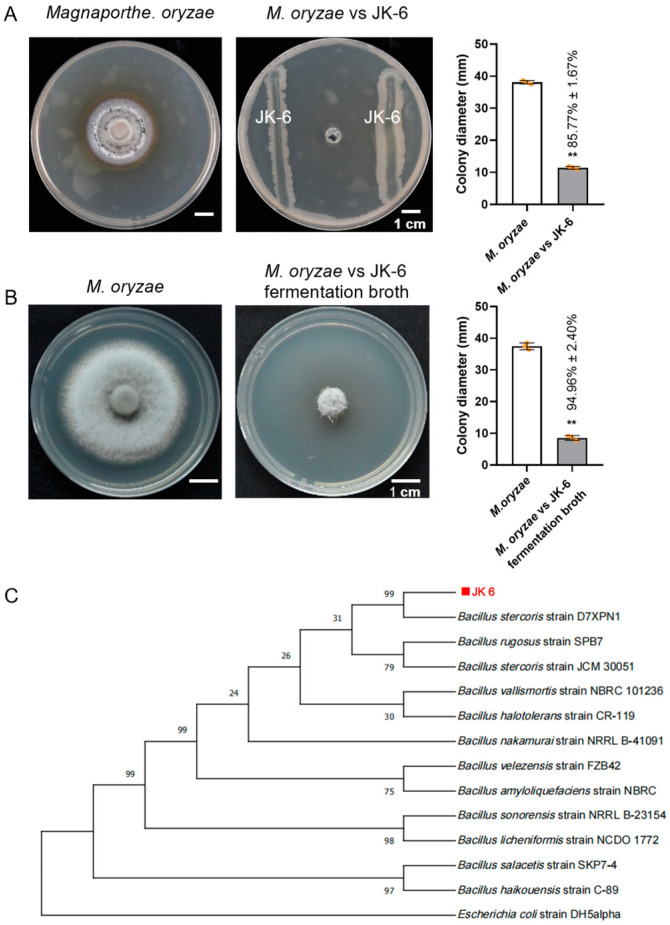
Identification of strain JK-6. (**A**) Antagonistic ability of JK-6 against *M. oryzae* grown on PDA medium. After incubation at 28 °C for six days, the colonies were photographed. (**B**) Inhibitory effects of 10% (*v*/*v*) JK-6 fermentation broth on mycelial growth of *M. oryzae*. JK-6 was cultured in liquid PDB medium, and its cell-free fermentation broth was collected by filtration. Subsequently, 10% (*v*/*v*) fermentation broth was incorporated into CM plates. *M. oryzae* was inoculated onto this plate to assess the inhibitory effect of the fermentation broth on *M. oryzae* growth. Scale bars: 1 cm. Data are presented as mean ± s.d., *n* = 3 biologically independent plate colonies. Orange bullets represent colony diameters. The inhibition rates are shown on the bar as mean ± s.d. Petri dishes are 9 cm in diameter for (**A**) and 6 cm in diameter for (**B**). Double asterisks (**) indicate highly significant differences (two-sided Student’s *t*-test, *p* ≤ 0.01). (**C**) Phylogenetic tree containing strain JK-6 based on the 16S rRNA sequence. Strain JK-6 is highlighted in red. The tree was constructed using the neighbor-joining method in the MEGA software (version: 12). The level of bootstrap support (1000 repetitions) is indicated at all nodes. Experiments were repeated three times with similar results.

Rhizosphere soil is an important source of biocontrol agents [30,31]. To obtain bacteria with potential antifungal activity for biocontrol of crop fungal diseases, we collected rhizosphere soil samples from rice paddies and isolated bacterial strains. We then used a cross-culture assay to evaluate their ability to inhibit the growth of the rice blast fungus *M. oryzae* Guy11 [32]. Among isolated bacterial strains, a bacterium designated JK-6 exhibited superior antifungal activity by strongly inhibiting mycelial growth of Guy11 in the cross-culture assay (Figure 1A). In addition, we collected PDB culture filtrates of this bacterium to characterize its fermentation broth in terms of inhibitory activity against fungal growth. Under standard conditions in CM agar medium, the colony diameter of *M. oryzae* Guy11 was 37.46 ± 1.06 mm; when grown in CM agar medium supplemented with 10% bacterial fermentation broth, the growth diameter of Guy11 was reduced to 8.55 ± 0.77 mm, corresponding to an inhibition rate of 94.96% ± 2.40% (Figure 1B). Its significant antagonistic activity against *M. oryzae* indicates that the isolated bacterium can be employed as a biocontrol agent.

We next examined the morphological properties and molecular identity of the JK-6 strain. The bacterial colonies appeared milky white with irregular serrated edges and exhibited a moist, viscous surface when grown on potato dextrose agar (PDA) plates (Supplementary Figure S1A). Scanning electron microscopy revealed a rod-like morphology of the JK-6 bacterium (Figure S1B). Phylogenetic analysis of the 16S rRNA sequence demonstrated that strain JK-6 showed the highest homology with *Bacillus stercoris* D7XPN1, with 99% sequence similarity (Figure 1C). These results indicate that JK-6 belongs to the genus *B. stercoris*.

### 3.2. Broad-Spectrum Antifungal Activity of JK-6

**Figure 2:**
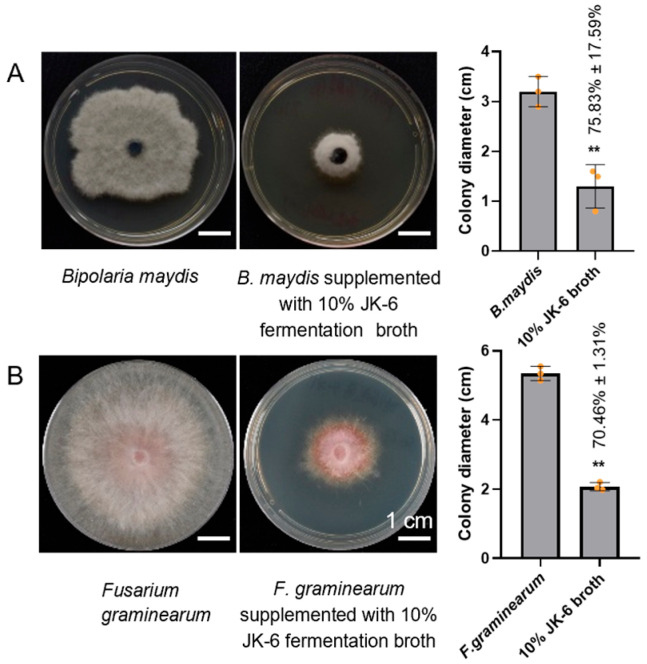
Antifungal activity of JK-6 fermentation broth against different phytopathogenic fungi. JK-6 was cultured in liquid PDB medium, and its cell-free fermentation broth was collected by filtration. Subsequently, 10% (*v*/*v*) fermentation broth was incorporated into PDA plates. Fungal pathogens *B. maydis* (**A**) and *F. graminearum* (**B**) were inoculated onto these plates to assess the inhibitory effect of the fermentation broth on fungal growth. After incubation at 28 °C for 5 days, the colonies were photographed, and their diameters were measured and statistically analyzed. Scale bars: 1 cm. Data are presented as mean ± s.d., *n* = 3 biologically independent plate colonies. Orange bullets represent colony diameters. The inhibition rates are shown on the bar as mean ± s.d. Double asterisks (**) indicate highly significant differences (two-sided Student’s *t*-test, *p* ≤ 0.01). Experiments were repeated three times with similar results.

We conducted a plate growth assay using medium supplemented with the fermentation broth of JK-6 to determine whether this *B. stercoris* strain harbors broad-spectrum antifungal activity. After JK-6 was grown in PDB liquid medium for three days, the fermentation broth was filtered to remove the bacteria before the assay. The test pathogens included *B. maydis* (the causal agent of southern corn leaf blight) and *F. graminearum* (the pathogen of wheat head blight). As shown in Figure 2, 10% PDB fermentation broth of *B. stercoris* JK-6 effectively inhibited the growth of *B. maydis* and *F. graminearum*, with inhibition rates of 75.83% ± 17.59% and 70.46% ± 1.31%, respectively. Hence, *B. stercoris* strain JK-6 exhibits broad-spectrum antifungal activity and merits further study.

### 3.3. Secondary Metabolite Gene Clusters of JK-6 by Genome Analysis

**Figure 3:**
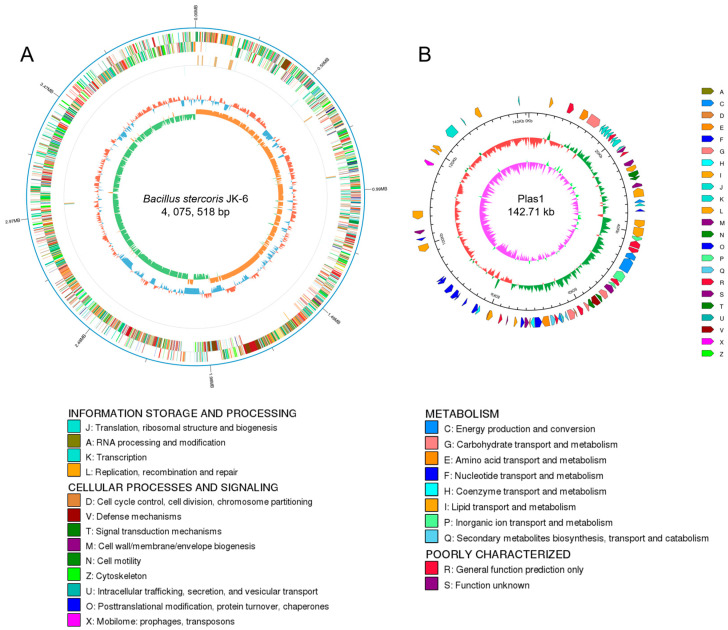
Circular genome and plasmid map of *B. stercoris* JK-6. (**A**) From the outermost to the innermost layers: Ring 1 represents the genomic sequence position coordinates, ring 2 the COG classifications of protein-coding genes on the forward and reverse strands, ring 3 the distribution of ncRNAs, ring 4 the GC content, and ring 5 the GC skew. (**B**) From the outermost to the innermost layers: Ring 1 represents the COG functional annotation categories of genes (with arrows indicating positive strand coding in a clockwise direction), ring 2 the genomic sequence position coordinates, ring 3 the GC content, and ring 4 the GC skew.

To characterize the molecular basis of the biocontrol properties of *B. stercoris* strain JK-6, we employed a combination of second- and third-generation sequencing to obtain the whole-genome sequence of JK-6 (Figure 3). The full genome sequence of JK-6 has been deposited in the GSA of the China National Center for Bioinformation (https://ngdc.cncb.ac.cn, accessed on 17 June 2026) under accession code PRJCA050265. Genomic analysis showed that JK-6 possesses one chromosome and one plasmid. The chromosome includes 4,075,518 bp with 43.67% G + C content (Figure 3A), and the plasmid includes 142,709 bp with 37.68% G + C content (Figure 3B). The GeneMarkS bioinformatic tool predicted that its sequenced genome consists of 4360 coding genes with a total length of 3,753,129 bp, accounting for 88.97% of the genome. Functional profiling via Gene Ontology (GO) categorized 13,757 annotated genes into biological processes, cellular components, and molecular functions (Figure S2A), and subsequent KEGG enrichment mapped 4002 genes to six major KEGG pathway categories (Figure S2B). Detailed classification data are shown in Figure S2 in the Supplementary Materials.

Furthermore, the antiSMASH 8.0 software [25] predicted that the JK-6 genome harbors 12 secondary metabolite gene clusters (Table 1). Four non-ribosomal peptide synthetase (NRPS) gene clusters were identified from the 12 gene clusters, with one gene cluster exhibiting 82% similarity to the surfactin-encoding cluster, one exhibiting 25% similarity to the pelgipeptin-encoding cluster, and the remaining two exhibiting 100% similarity to clusters involved in fengycin and bacillibactin synthesis. Additionally, two polyketide synthase (PKS) gene clusters were identified, with one displaying 100% similarity to trans-acyl transferase PKS (transAT-PKS), involved in bacillaene synthesis, and the other displaying 16% similarity to the Type III PKS (T3PKS) gene cluster, involved in 1-carbapen-2-em-3-carboxylic acid synthesis. Furthermore, there were two terpene gene clusters, one ranthipeptide gene cluster, one sactipeptide gene cluster, one epipeptide gene cluster, and one bacilysin-encoding cluster. Most of these genes are implicated in antimicrobial production and are conserved across diverse *B. stercoris* strains.

### 3.4. Production of Secondary Metabolite Surfactin by JK-6

**Figure 4:**
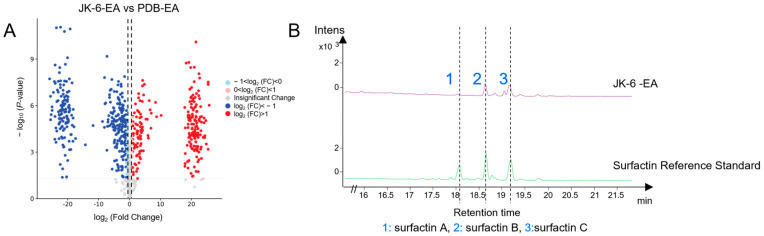
(**A**) A volcano plot comparing the JK-6-EA vs. PDB-EA groups, employing a screening criterion of fold-change ≥ 1.5 or fold-change ≤ 1/1.5, along with a *p*-value < 0.05. EA: ethyl acetate. JK-6-EA: ethyl acetate extract of fermentation broth of strain JK-6; PDB-EA: ethyl acetate extract of blank PDB medium (control group). The vertical dashed lines indicate log_2_(1/1.5) and log_2_(1.5). In the plot, red dots represent upregulated metabolites, while blue dots signify downregulated metabolites. Fold change is abbreviated as FC. (**B**) HPLC chromatogram illustrating the identification of surfactins in the ethyl acetate extract of JK-6. Experiments were repeated three times with similar results.

Genome sequencing revealed that the genome of strain JK-6 contains genes associated with the lipopeptide compound surfactin, indicating that this bacterium can produce surfactin. The fermentation broth of JK-6 cultured in PDB medium was extracted with ethyl acetate, and the crude extract was analyzed to examine the production of surfactin by JK-6. The PDB liquid medium without JK-6 inoculation was used as a control and similarly extracted. Metabolomics analysis of the crude extracts through mass spectrometry identified 519 significantly different metabolites between the extract of the JK-6 fermentation broth and the control extract, with 209 upregulated and 310 downregulated metabolites in the JK-6 extract (Figure 4A). Mass spectrometry analysis in positive ion mode detected 1198-fold, 63-fold, and 12-fold higher levels of surfactins A, B, and C in the JK-6 extract, respectively, compared with those in the control extract (Supplementary Table S1). Consistently, ultra-performance liquid chromatography–tandem-mass spectrometry (UPLC–MS/MS) analysis showed that surfactins A, B, and C were indeed present in the JK-6 extract, with retention times of 18.2 min, 18.7 min, and 19.2 min, respectively (Figure 4B).

### 3.5. Broad Antifungal Activity of Surfactin

**Figure 5:**
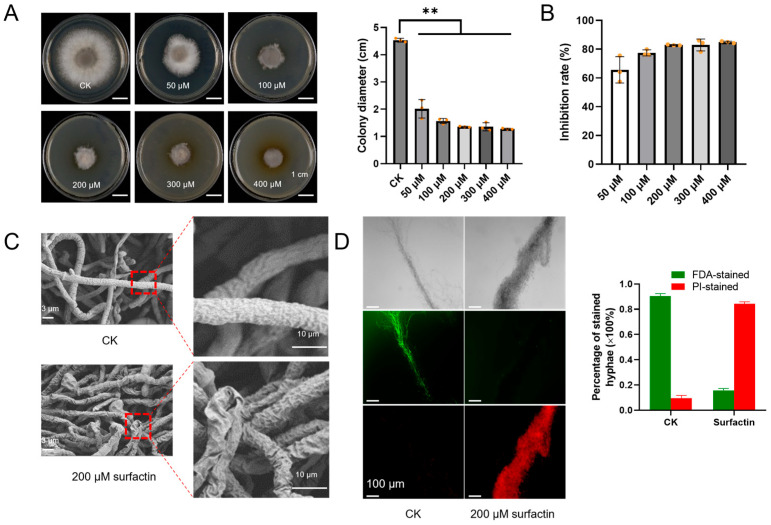
Effects of surfactin on growth of *M. oryzae*. (**A**) Plate colony of *M. oryzae* grown on agar CM supplemented with surfactin at concentrations of 50 μM, 100 μM, 200 μM, 300 μM, and 400 μM. Scale bars: 1 cm. (**B**) Inhibition rates of *M. oryzae* grown on agar CM supplemented with different concentrations of surfactin. (**C**) SEM examination of mycelium of *M. oryzae* grown without surfactin (CK) or exposed to 200 μM surfactin. Scale bars: 3 μm (**left panels**) and 10 μm (**right magnified panels**). (**D**) FDA-PI double staining of mycelium of *M. oryzae* grown without surfactin (CK) or exposed to 200 μM surfactin. Green fluorescence represents FDA staining, and red fluorescence denotes PI staining. Scale bars: 100 μm. Data are presented as mean ± s.d., *n*  =  biologically independent samples: *n*  =  3 plate colonies (**A**); *n*  =  3 fungal hyphae of *M. oryzae* (**D**). Orange bullets represent colony diameters for (**A**) and inhibition rates for (**B**). Double asterisks (**) indicate highly significant differences (two-sided Student’s *t*-test, *p* ≤ 0.01). Experiments were repeated at least two (**D**) or three times (**A**–**C**) with similar results.

To quantify the inhibitory activity of surfactin against *M. oryzae*, we conducted an antibiotic agar plate experiment by adding various concentrations of surfactin to CM plates. Measurement of plate colony diameter showed that surfactin inhibited fungal growth by 65.66% ± 17.59%, 77.44% ± 2.13%, 82.90% ± 0.37%, 82.88% ± 4.12%, and 84.90% ± 0.65% at concentrations of 50 μM, 100 μM, 200 μM, 300 μM, and 400 μM, respectively (Figure 5A,B). This result suggests that the inhibitory effect reached saturation at a concentration of 200 μM. Accordingly, we selected 200 μM surfactin for subsequent experiments. Scanning electron microscopy (SEM) analysis showed that the mycelium of *M. oryzae* exposed to 200 μM surfactin exhibited a shriveled and wilted morphology (Figure 5C) compared to the unexposed control mycelium (CK). Additionally, FDA-PI double staining showed a markedly higher percentage of red-stained mycelia (indicating membrane damage) in the 200 μM surfactin group relative to the control (Figure 5D), confirming that surfactin damages the fungal cell membrane. These results indicate that surfactin significantly impaired the growth of the rice blast fungus.

**Figure 6:**
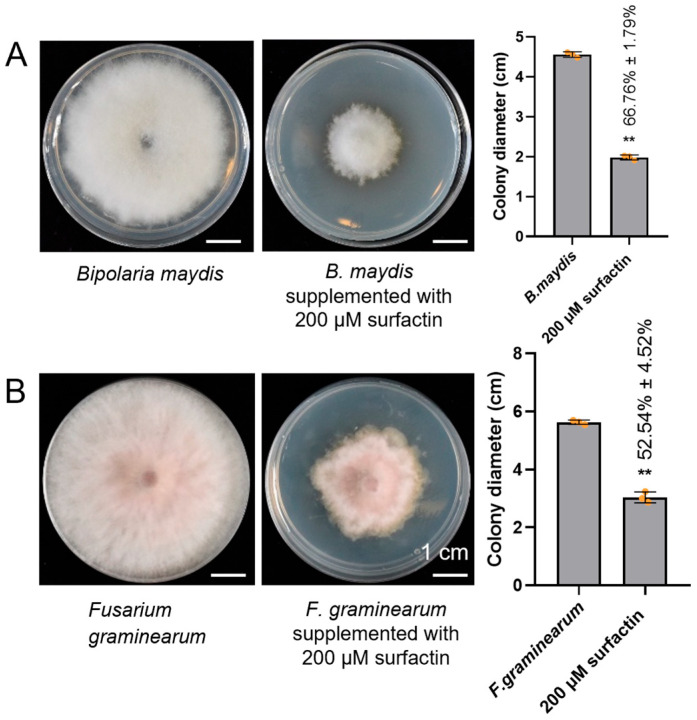
Antifungal activity of 200 μM surfactin against phytopathogenic fungi. PDA plates were supplemented with or without 200 μM surfactin and then used to measure the growth of various fungal pathogens, viz., *B. maydis* (**A**) and *F. graminearum* (**B**). Scale bars: 1 cm. Petri dishes are 6 cm in diameter. Data are presented as mean ± s.d., *n* = 3 biologically independent plate colonies. Orange bullets represent colony diameters. The inhibition rates are shown on the bar as mean ± s.d. Double asterisks (**) indicate highly significant differences (two-sided Student’s *t*-test, *p* ≤ 0.01). Experiments were repeated three times with similar results.

We further tested the potential broad-spectrum antifungal activity of surfactin against other fungal pathogens, including *B. maydis* and *F. graminearum*. As illustrated in Figure 6, 200 μM surfactin effectively inhibited the growth of *B. maydis* and *F. graminearum*, with inhibition rates of 66.76% ± 1.79% and 52.54% ± 4.52%, respectively (Figure 6). Hence, surfactin can be employed as an effective antifungal agent with broad-spectrum antifungal activity.

### 3.6. Molecular Mechanism of Surfactin’s Inhibition of M. oryzae

**Figure 7:**
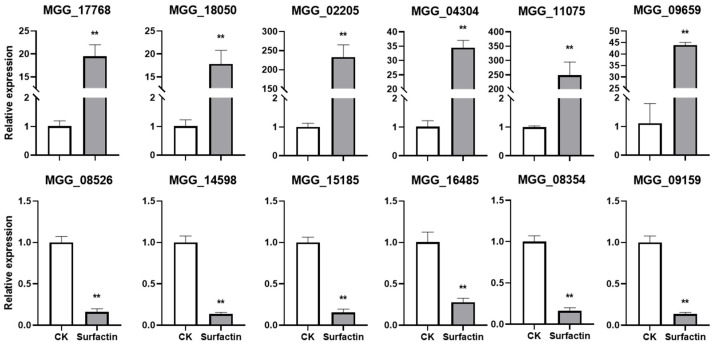
Analysis of 12 randomly selected DEGs by qRT-PCR. Total RNA was extracted from mycelia of *M. oryzae* treated with or without 200 μM surfactin for 24 h and reverse-transcribed into cDNA for qRT-PCR analysis. White bars correspond to the control group (CK), and gray bars show the relative expression levels in M. oryzae mycelia after treatment with 200 μM surfactin.Values are mean ± s.d. *n* = 3 biologically independent mycelial RNA. Statistical analysis was performed using two-sided Student’s *t*-test. **, *p* ≤ 0.01. Experiments were repeated three times with similar results.

We next conducted a transcriptomic analysis to further elucidate the molecular events underlying surfactin’s inhibition of *M. oryzae*. RNA samples were collected from the mycelium after 48 h of treatment with surfactin and control ddH_2_O. Pearson’s correlation coefficients ranged from 0.82 to 1, indicating high consistency among the three independent replicates (Supplementary Figure S3). Genes with at least 2-fold higher or lower expression levels in the surfactin treatment compared to the control treatment were identified as differentially expressed genes (DEGs). Only DEGs detected in all three independent biological replicates were selected for further analysis. We randomly selected 12 DEGs for qRT-PCR analysis and validated the reliability of the transcriptome data (Figure 7).

**Figure 8:**
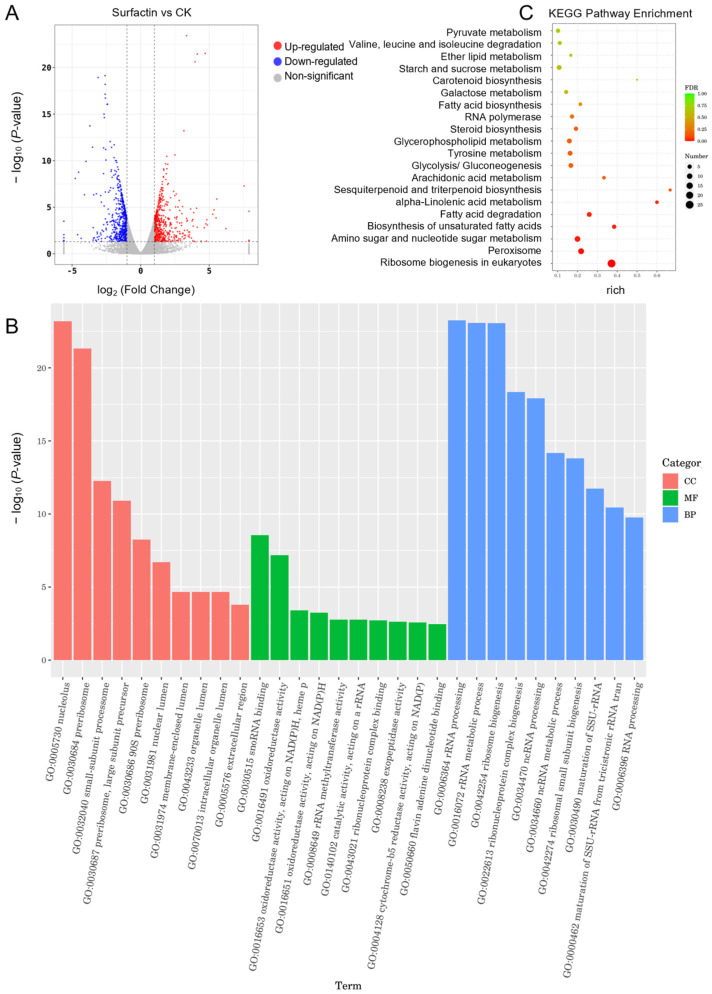
Categorization of DEGs according to GO and KEGG analyses. (**A**) Volcano plot of DEGs identified in *M. oryzae* mycelia treated with 200 μM surfactin for 24 h, compared to the untreated control. Red, blue, and gray dots represent upregulated, downregulated, and non-significant genes, respectively. The two vertical dashed lines indicate the threshold for a 2-fold change in expression, while the horizontal dashed line represents the significance threshold of *p* = 0.05. (**B**) GO enrichment analysis of DEGs, categorized into cellular components (CC, red), molecular functions (MF, green), and biological processes (BP, blue). The *y*-axis shows the −log_10_(*p*-value) of each GO term. (**C**) Top 20 significantly enriched KEGG pathways of DEGs. The ordinate represents the KEGG pathway, and the abscissa represents the Rich factor. The larger the Rich factor, the greater the enrichment. The larger the point, the greater the number of differential genes enriched in the pathway. The size of the dots indicates the number of DEGs enriched in the pathway, and the color gradient represents the false discovery rate (FDR) value, where the redder the dots, the more significant the enrichment.

In total, we identified 1056 DEGs—501 upregulated and 555 downregulated—in *M. oryzae* exposed to surfactin (Figure 8A). Based on Gene Ontology (GO) functional annotation, DEGs were categorized into three classes: cellular components, molecular functions, and biological processes. In the cellular component category, the most enriched terms were nucleus, preribosome, small-subunit processome, and large-subunit precursor. The most enriched terms of molecular function were snoRNA binding; oxidoreductase activity; oxidoreductase activity, acting on NAD(P)H heme protein; and oxidoreductase activity, acting on NAD(P)H. In addition, the enriched terms of biological processes covered rRNA processing, ribosome biogenesis, rRNA metabolic process, and ribonucleoprotein complex biogenesis (Figure 8B).

Kyoto Encyclopedia of Genes and Genomes (KEGG) pathway enrichment analysis revealed that the DEGs were mainly involved in tyrosine metabolism, glycolysis/gluconeogenesis, arachidonic acid metabolism, sesquiterpenoid and triterpenoid biosynthesis, alpha-linolenic acid metabolism, fatty acid degradation, biosynthesis of unsaturated fatty acids, amino sugar and nucleotide sugar metabolism, peroxisome function, and ribosome biogenesis in eukaryotes (Figure 8C). Among the DEGs, significantly downregulated genes were associated with endoglucanases, glycoside hydrolases, xylanases, catalases, superoxide dismutase, histone acetyltransferases, and DNA repair proteins (Table S3). In contrast, upregulated genes were enriched in retinol dehydrogenase, cytochrome P450, ABC transporters, and MFS transporters (Table S4). Together, these results suggest that surfactin compromises fungal growth by affecting the expression of a vast range of genes associated with diverse metabolic processes and signaling pathways important for fungal development.

## 4. Discussion

In recent years, biological control has emerged as a highly effective, safe, and environmentally friendly strategy for controlling plant disease agents. Among the various biological control bacteria, *Bacillus* spp. are believed to be effective [33], but their use in biocontrol remains insufficiently explored and their biocontrol metabolites remain poorly characterized, limiting their application in practical crop production. In this study, a *Bacillus* strain characterized as *B. stercoris* JK-6 exhibited potent antifungal activity against several phytopathogens, especially *M. oryzae*. Many *Bacillus* strains, including *B. subtilis*, *Bacillus amyloliquefaciens*, and *Bacillus velezensis*, have inhibitory activities against mycelial growth or spore germination [34,35,36]. However, few *B. stercoris* isolates have exhibited antagonistic activity against *M. oryzae*. Our study found that *B. stercoris* JK-6 significantly inhibited the mycelial growth of *M. oryzae*, offering a novel approach and a robust basis for rice blast management.

Analysis of the JK-6 genome revealed 12 gene clusters linked to secondary metabolite biosynthesis, covering well-known antifungal BGCs responsible for surfactin [37,38], fengycin [39,40,41], bacillaene, bacillibactin [42], bacilysin, and subtilosin A biosynthesis. Consistent with prior genomic reports on other *B. stercoris* isolates [13,43], these conserved clusters imply the inherent capacity of this species to synthesize diverse lipopeptide antimicrobials. Combining metabolomics analysis and HPLC-MS/MS results, we definitively validated that surfactins A, B, and C were produced by strain JK-6. The literature confirms the broad-spectrum antifungal performance of lipopeptides derived from various Bacillus strains [44], but few reports delve into the specific transcriptional-level antifungal mechanisms of the surfactins produced by these strains against rice blast fungus. Our transcriptomic data illustrate that surfactin treatment drastically reshaped the global transcriptional profile of *M. oryzae*, resulting in differential expression of 1056 genes, comprising 501 upregulated and 555 downregulated genes.

Downregulated functional genes mainly clustered into three pivotal physiological modules of *M. oryzae*: cell wall-degrading hydrolases, antioxidant enzymes, and epigenetic/DNA repair-related proteins. Cell wall hydrolases are well-documented to be essential for development and pathogenic virulence of diverse phytopathogenic fungi [45,46,47,48,49,50]. Consistently, our transcriptomic data revealed that a battery of genes encoding the hydrolytic enzymes was downregulated after surfactin treatment, implying that growth retardation of treated *M. oryzae* is tightly associated with transcriptional suppression of cell wall-degrading hydrolase genes. Peroxisomes are monolayer organelles commonly found in eukaryotes and contain oxidase, catalase, and peroxidase [51]. Catalase is a peroxidase marker enzyme; its main function is to hydrolyze the cytotoxic substance H_2_O_2_ produced in oxidase-catalyzed redox reactions [52]. Seven downregulated genes were significantly enriched in the “Peroxidase” pathway: four catalase genes (MGG_09834, MGG_04337, MGG_10061, and MGG_06442) and three superoxide dismutase genes (MGG_00212, MGG_00491, and MGG_02625). These changes may not only affect the oxidation of toxic substances, such as formic acid and phenol, but also result in H_2_O_2_ accumulation, leading to cell damage.

We detected two histone acetyltransferases (GCN5: MGG_03677 and MGG_11716) and several histone deacetylases (HDA1: MGG_01076; RPD3: MGG_05857; PHD1: MGG_01633; HOS3: MGG_06043) that were downregulated upon surfactin exposure. Histone acetyltransferases (HATs) and histone deacetylases (HDACs) are critical for growth, development, and DNA damage repair in phytopathogenic fungi [53]. Previous functional research has validated that *MoHDA1* regulates asexual development and pathogenicity of *M. oryzae* [54], while knockout of GCN5, HDA1, and RPD3 in *Alternaria alternata* impairs fungal growth, conidiation, virulence, and tolerance against DNA-damaging stressors [53]. In addition, a battery of DNA repair genes was downregulated in our transcriptomic analysis, including MGG_06208, MGG_04561, MGG_05032, MGG_06470, MGG_11387, MGG_05239, MGG_15576, MGG_07015, MGG_06094, MGG_03549, MGG_04175, MGG_02804, MGG_11047, MGG_08585, and MGG_07014. Collectively, these results suggest that the surfactins may inhibit growth and DNA damage repair in *M. oryzae* by downregulating the expression of genes related to histone acetyltransferase, histone deacetylase, and DNA repair proteins.

Furthermore, the pathogen activated a series of intrinsic detoxification and stress-adaptive transcriptional responses upon surfactin exposure. Genes encoding retinol dehydrogenase, cytochrome P450, oxidoreductase, ABC transporters, and major facilitator superfamily (MFS) transporters were significantly upregulated in surfactin-treated *M. oryzae*. Retinol dehydrogenases participate in cell wall dextran synthesis, and these retinol dehydrogenase genes (MGG_04304, MGG_10913, MGG_06534, MGG_10613, and MGG_07491) may be involved in the stress repair process after cell wall destruction is induced by surfactin [55], while cytochrome P450, oxidoreductase, and ABC and MFS transporters are canonical detoxification components that mediate efflux and metabolic degradation of exogenous toxic compounds in fungi [56,57,58,59,60,61,62]. In our transcriptomic analysis, a battery of cytochrome P450 genes was upregulated, including MGG_00832, MGG_04349, MGG_04628, MGG_07551, and MGG_14591. One oxidoreductase (MGG_10710) and two dehydrogenase/reductase SDR family members (MGG_08349 and MGG_00426) showed the same regulation trend. In addition, two ABC transporter genes (MGG_13624, MGG_07848) and one MFS transporter gene (MGG_00416) were upregulated. These upregulated transcripts reflect the conserved defensive cascade of *M. oryzae* against lipopeptide stress, which partially alleviates the inhibitory damage triggered by surfactin.

Considering the results of the above analyses, we speculate that the following response occurs in *M. oryzae* upon exposure to surfactins produced by *B. stercoris* JK-6: Surfactins affect endoglucanase, glycoside hydrolase, and xylanase, which are responsible for the development and virulence of *M. oryzae*. The catalase and superoxide dismutase genes responsible for scavenging oxygen free radicals in *M. oryzae* cells are downregulated, and accumulation of toxic substances leads to cell damage. In addition, a battery of DNA repair genes is downregulated. Unsurprisingly, *M. oryzae* cells produce multiple responses to counteract stress. Retinol dehydrogenase genes, involved in dextran biosynthesis and the synthesis and repair of cell walls and cell membranes, are upregulated. Additionally, a battery of cytochrome P450 genes responsible for detoxification of *M. oryzae* is upregulated. The oxidoreductase and ABC transporter genes are upregulated to enhance antioxidant function and to excrete extracellular substances. In future work, we will perform gene knockout and overexpression analyses of these key genes to further elucidate the inhibitory mechanism of surfactin against *M. oryzae*, laying a theoretical foundation for deciphering the biocontrol mode of action of *B. stercoris* JK-6.

Despite the promising in vitro antifungal results obtained in this study, several notable limitations should be acknowledged to objectively define the boundary of our conclusions and guide follow-up research. All antagonistic bioassays of the JK-6 strain and surfactin were performed exclusively under in vitro pure culture conditions on artificial agar medium; we have not validated their control efficacy in greenhouse potted rice or field cropping environments. Abiotic factors, including soil pH, indigenous rhizosphere microbiota fluctuation, temperature, and rainfall in natural field conditions, would markedly alter the colonization efficiency of JK-6 and the stability of secreted surfactin; hence, the in vitro inhibitory data cannot be directly extrapolated to practical field disease management. Therefore, it will be necessary and interesting to conduct greenhouse and field trials to evaluate the practical biocontrol efficiency of JK-6 fermentation broth and formulated surfactin against natural rice blast infection. These follow-up works will solidify the translational application potential of *B. stercoris* JK-6 in crop disease biocontrol.

## 5. Conclusions

In this study, the bacterial strain JK-6 was isolated and identified as *B. stercoris* through 16S rRNA gene and whole-genome sequencing. *B. stercoris* JK-6 and the surfactin that it produces showed significant antagonistic activity against phytopathogens. Transcriptomic and metabolomic analyses were conducted to explore the underlying antagonistic mechanism of surfactin, and the results revealed several pathways that may play important roles in *M. oryzae* detoxification. The results may provide a theoretical basis for the potential application of *B. stercoris* JK-6 for future plant protection.

## Figures and Tables

**Table 1:** Gene clusters linked to secondary metabolite biosynthesis in JK-6.

Gene Clusters	Types	Genome Locations	Most Similar Known Clusters	Similarity
Chromosome				
Cluster 1	Ranthipeptide, sactipeptide	203,445–225,523	Sporulation killing factor	100%
Cluster 2	NRPS	356,578–420,013	Surfactin	82%
Cluster 3	Terpene	1,128,210–1,149,016	-	-
Cluster 4	TransAT-PKS, PKS-like, T3PKS, NRPS	1,742,592–1,847,853	Bacillaene	100%
Cluster 5	NRPS, betalactone	1,925,698–2,002,451	Fengycin	100%
Cluster 6	T3PKS	2,137,805–2,178,902	1-Carbapen-2-em-3-carboxylic acid	16%
Cluster 7	NRPS, ladderane	2,308,931–2,366,338	Pelgipeptin	25%
Cluster 8	NRP-metallophore, NRPS	3,107,895–3,159,672	Bacillibactin	100%
Cluster 9	Sactipeptide	3,693,057–3,714,668	Subtilosin A	100%
Cluster 10	Other	3,717,664–3,759,082	Bacilysin	100%
Cluster 11	Epipeptide	3,969,957–3,991,655	Thailanstain A	10%
Plasmid				
Cluster 12	terpene	29,016–50,914	-	-

Note: “-” denotes none.

## Data Availability

The genomic sequences and transcriptomic data of strain JK-6 have been submitted to the China National Center for Bioinformation (CNCB) (https://ngdc.cncb.ac.cn/bioproject/browse/PRJCA050265, accessed 17 June 2026) under the accession number PRJCA050265. The untargeted metabolomics data have been deposited in the OMIX database of CNCB (https://ngdc.cncb.ac.cn/bioproject/browse/PRJCA066201, accessed 8 June 2026) under the accession number PRJCA066201. All relevant data are available in the main text and Supplementary Materials. Further inquiries can be directed to the corresponding authors.

## Supplementary Materials

The following supporting information can be downloaded at https://www.mdpi.com/article/10.3390/jof12070467/s1: Figure S1: Identification of strain JK-6; Figure S2: Gene function classification; Figure S3: Pearson correlation coefficients among samples; Figure S4. Dose–response and IC_50_ analysis of surfactin against *M. oryzae;* Figure S5: Chromatogram corresponding to LC-MS of mass spectra of the surfactin; Table S1: Surfactins in the ethyl acetate extract of strain JK-6; Table S2: Primers used for qRT-PCR in this study; Table S3: Down-regulated functional genes in *M. oryzae* exposed to surfactin; Table S4: Up-regulated functional genes in *M. oryzae* exposed to surfactin.
