# Supplementary material for: Characterization of Bacillus stercoris JK-6 as an Antifungal Agent Against Crop Fungal Diseases
Source: J Fungi (Basel). 2026 Jun 25;12(7):467. doi: 10.3390/jof12070467 (PMC13412506; doi:10.3390/jof12070467)
Supplement: Supplementary file 1 [file jof-12-00467-s001.zip › jof-4297968-supplementary.pdf]

Characterization of *Bacillus stercoris* JK-6 as an Antifungal Agent Against Crop Fungal Diseases

Qing Ouyang#, Jiazheng Wang#, Xiangyan Liu, Siyang Wang, Zirui Chen, Huabin Zhou, Xiaolin Chen, Xiang Lu, Qing Xiong, Jia Su, Tuo Qi, Xuewei Chen* and Min He*

SUPPLEMENTAL DATA

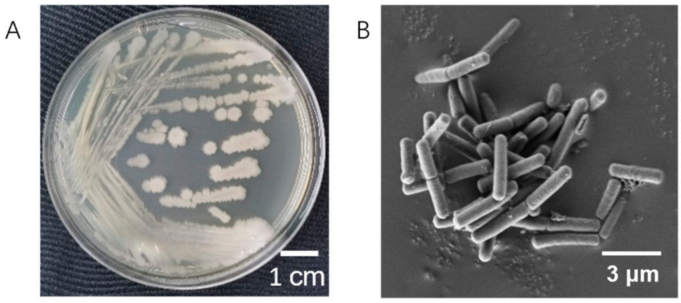

Figure S1. Identification of strain JK-6. (A) Colony morphology observation of *Bacillus stercoris* JK-6, bar=1 cm. (B) Scanning electron microscopy graph of strain JK-6, bar=3 μm. Experiments were repeated three times with similar results.

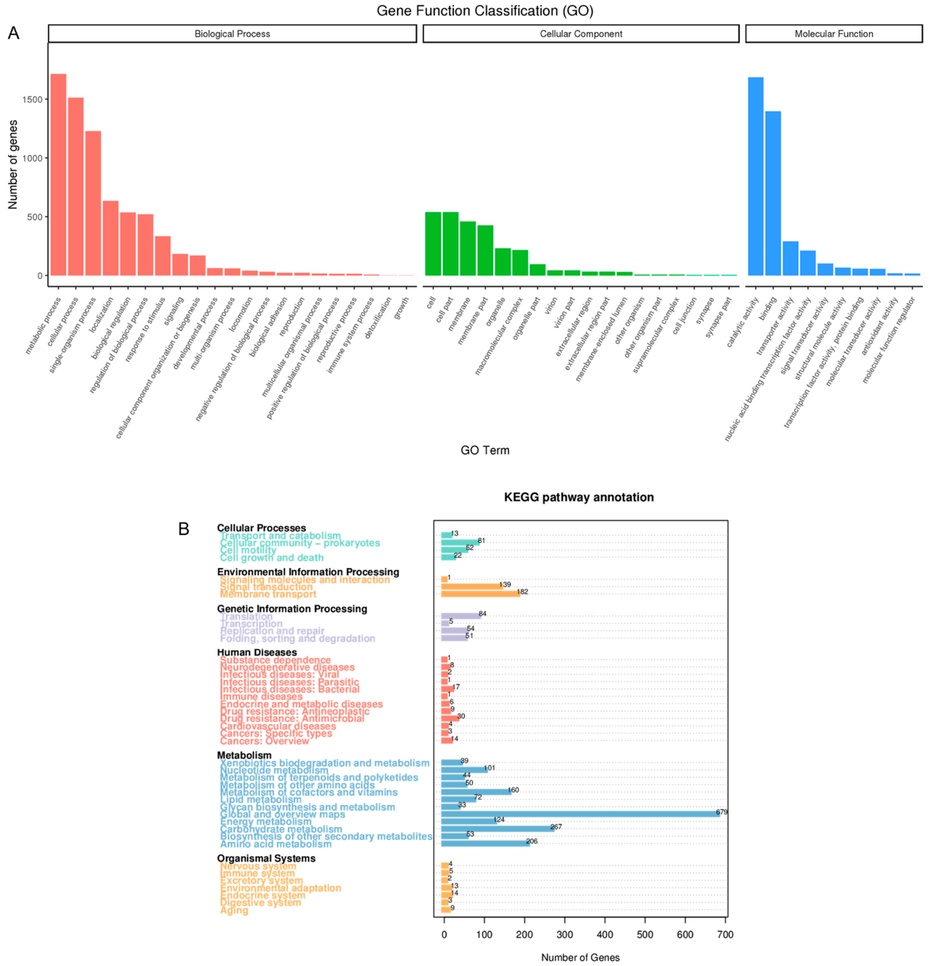

Figure S2. Gene Function Classification. (A) and KEGG pathway annotation (B) of *B. stercoris* JK-6 genenome. A. The x-axis represents the GO terms at the next level beneath the three main categories of Gene Ontology, while the y-axis indicates the number of genes annotated to each term, including its sub-terms. The three different classifications correspond to the three fundamental categories of GO, namely biological process, cellular component, and molecular function, from left to right. B. The numbers on the bar chart represent the number of annotated genes, while the other axis displays the codes for each functional category at level 1 in the database, with explanations provided in the corresponding legend.

Supplementary Discription for Figure S2:

The functional landscape of *B. stercoris* strain JK-6 was delineated through Gene Ontology (GO) analysis, which assigned 13,757 protein-coding genes to three primary functional categories: Biological Process, Cellular Component, and Molecular Function (Figure S2A). Within the Biological Process category, the most abundantly represented terms were metabolic process (1,714 genes), cellular process (1,514 genes), and single-organism process (1,229 genes). These findings underscore the strain's robust metabolic capabilities and fundamental cellular activities, which are central to its survival and adaptation. For Cellular Component, the dominant terms were cell (538 genes), cell part (538 genes), and membrane (459 genes), consistent with the prokaryotic cellular architecture and the importance of membrane-associated functions. In Molecular Function, catalytic activity (1,686 genes) and

binding (1,398 genes) were the most highly enriched categories, reflecting the strain's extensive enzymatic repertoire and capacity for molecular interactions. To further dissect the metabolic and functional potential of *B. stercoris* strain JK-6, KEGG pathway analysis was performed, mapping 4,002 genes to six major KEGG pathway categories (Figure S2B). Within the Metabolism category, the most significantly enriched pathways were Global and overview maps (679 genes), Carbohydrate metabolism (267 genes), and Amino acid metabolism (206 genes). These pathways are indicative of the strain's robust capacity for energy production, carbon utilization, and biosynthesis of essential cellular building blocks. In Genetic Information Processing, genes were predominantly associated with Translation (84 genes), Folding, sorting and degradation (51 genes), and Replication and repair (54 genes). These pathways underscore the strain's ability to efficiently synthesize, process, and maintain its proteome and genome. For Environmental Information Processing, the most prominent pathway was membrane transport (182 genes), which is crucial for the uptake of essential nutrients, excretion of metabolic wastes, and translocation of signaling molecules across the cell membrane. In Cellular Processes, pathways related to Cell motility (52 genes) and Cellular community – prokaryotes (81 genes) were well-represented, indicating the strain's capacity for cell movement, chemotaxis toward favorable microenvironments, and formation of intercellular interactions within prokaryotic communities. Finally, genes involved in Human Diseases and Organismal Systems were also identified, albeit with lower gene counts. These annotations suggest potential functional overlaps with pathways relevant to host-microbe interactions and disease-related processes, warranting further investigation.

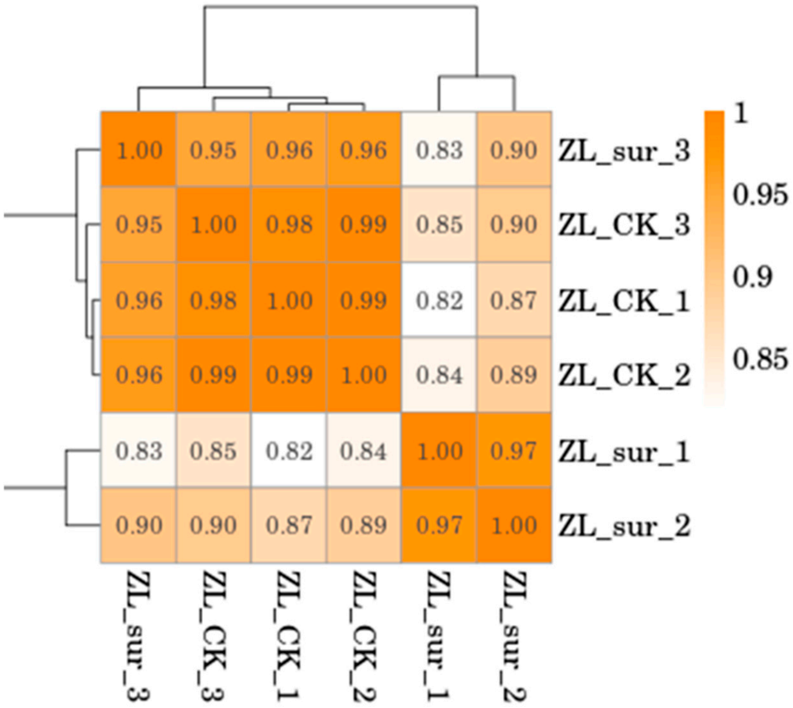

Figure S3. Pearson correlation coefficients among samples.

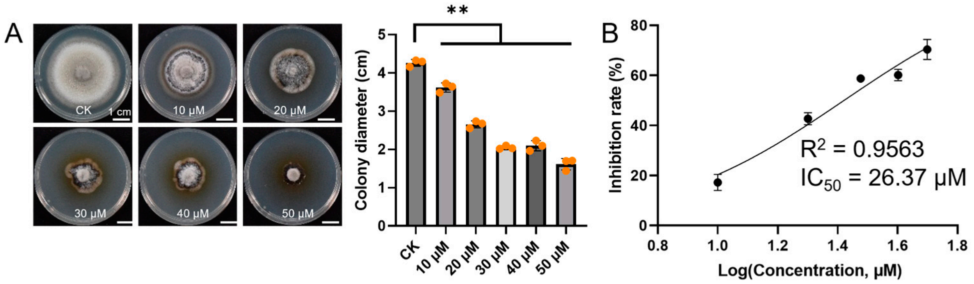

Figure S4. Dose-response and IC_{50} analysis of surfactin against *M. oryzae*. (A) Colony morphology and diameter under different surfactin concentrations. Scale bars: 1 cm. Petri dishes are 6 cm in diameter. Data are presented as mean ± s.d.. *n* = 3 biologically independent plate colonies. Double asterisks (**) indicate highly significant differences (two-sided Student's *t*-test, $p \leqslant 0.01$). Experiments were repeated three times with similar results. (B) Nonlinear regression curve of inhibition rate vs log(concentration), showing the calculated IC_{50} value.

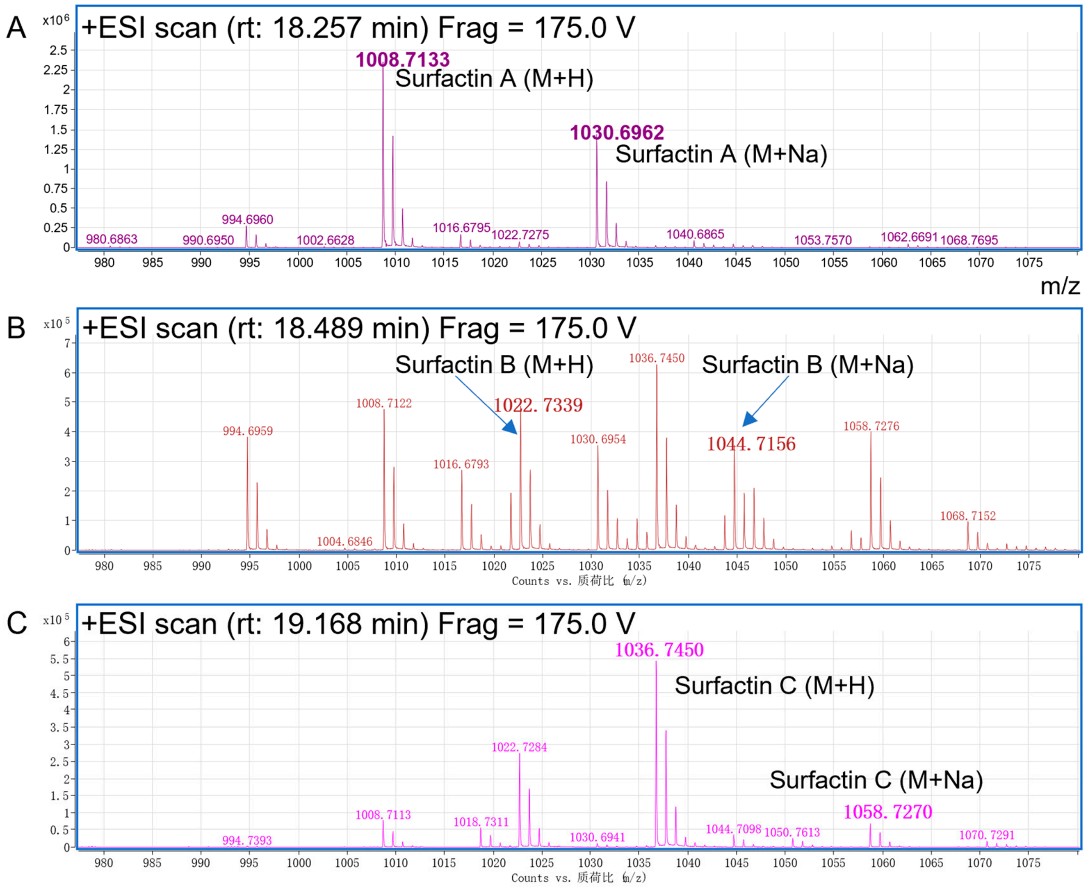

Figure S5. Chromatogram corresponding to LC-MS of mass spectra of the surfactin (A-C: m/z 1008.7133, 1022.7339, 1036.7450, respectively).

Table S1. Surfactins in the ethyl acetate extract of strain JK-6.

Compounds	JK-6-EA-1	JK-6-EA-2	JK-6-EA-3	PDB-EA-1	PDB-EA-2	PDB-EA-3	Fold change	Ion mode
Surfactin A	1046428830	952909033	969314282	1227261.85	635064.45	616485.3	1197.61104	+
Surfactin B	435202163	385590188	354187576	207.13638	11060148.8	7710703.59	62.5952907	+
Surfactin C	199192626	204422213	171254540	24441198.2	12149412.4	11729172.8	11.8971845	+

Table S2. Primers used for qRT-PCR in this study.

Gene name	Forward primer (5'-3')	Reverse primer (5'-3')
MoTubulin	CGTTGAGCCCTACAACGCTA	GGCCGAAACCAGGTAGTTCA
MGG_ 11768	GATGCGCCAATGCAACATGTCC	TCGCTGGTTACGTCTTGTTGCTC
MGG_ 18050	GCAAGAGTCCAGCAGCTCAATG	TCTCGTACACTACCCGTTCTGG
MGG_ 02205	AGGAGGAGAATTTGGCACACAAGC	TTAAGGAGCTGTCCGCAAACGG
MGG_ 04304	GCGGCGTCATTATAAACGTCAGC	ATGTTCCACTCTGGGCACATCTC
MGG_ 11075	CAACGACGCTATTGAATGG	ATCCGAAGTGGTGTGAAC
MGG_ 09659	CCATCAGGACGGAGAATACCA	GAGGCTGGAGTCTGCGAAT
MGG_ 08526	GGCAAGGAGTACAACATGAACGC	AAGCGGTTGTAGGCGGTAAAGG
MGG_ 14598	TGCACACTTCCATGTATGCCATC	GTTGGTGTGGTGCAACCAGAAG
MGG_ 15185	TCCTTCGTCGACCGCCAAGATTTC	TATCCTCTCTTACCGCAGCACTGG
MGG_ 16485	ACTTGCAGCGCGATAAAGGG	TGGTGAGATCGGTGTTCGGAAG
MGG_ 08354	CAACGTCCTCGACCAGAT	CAGGATGTCCTCGTCAGA
MGG_ 09159	CAGATGGTCAAGAACGAGAT	CGAAGTAGGTGATGTGGTAG

Table S3. Down-regulated functional genes in *M. oryzae* exposed to surfactin.

Gene ID	FoldChange (Sur vs CK)	log_2FoldChange	*P*-value	Description
MGG_08020	0.522603252	-0.936211994	0.000707387	Endoglucanase-4
MGG_06069	0.496193469	-1.011025349	0.003248071	Endoglucanase
MGG_08537	0.309911338	-1.69007256	0.012744305	Endoglucanase-1; Endoglucanase-1, variant
MGG_00677	0.561398526	-0.83290282	0.0530149	Endoglucanase-1
MGG_15430	0.472046025	-1.083000563	0.372269369	Endo-1,4-beta-xylanase A
MGG_11400	0.851850478	-0.231327873	0.44014407	Endo-1,4-beta-xylanase D
MGG_05464	0.920425915	-0.119626491	0.666078812	Endo-1,4-beta-xylanase
MGG_07955	0.785804983	-0.347756778	0.806259133	Endo-1,4-beta-xylanase 22
MGG_08424	0.434512118	-1.202531683	0.919540477	Endo-1,4-beta-xylanase I
MGG_13429	0.68308627	-0.549860301	0.248246912	Glycoside hydrolase
MGG_02096	0.66931858	-0.579235032	0.397068391	Glycoside hydrolase
MGG_04561	0.926417803	-0.110265117	0.888557143	DNA repair family protein
MGG_06470	0.68670258	-0.54224271	0.036075515	DNA repair helicase RAD25
MGG_05239	0.70355145	-0.507272164	0.138649263	DNA repair and recombination protein RAD26
MGG_07015	0.767225334	-0.382277736	0.187813611	DNA repair protein Rad7
MGG_06094	0.766941931	-0.382810746	0.242627541	DNA repair protein RAD5
MGG_02804	0.928005711	-0.107794412	0.671314061	DNA repair protein rad16
MGG_08585	0.979498262	-0.029885163	0.891041689	DNA repair and recombination protein rhm52
MGG_07014	0.999525188	-0.000685171	0.936085504	DNA repair protein RAD16
MGG_06043	0.839764556	-0.251943197	0.400227197	Histone deacetylase HOS3
MGG_09834	0.400120192	-1.32149466	1.5318E-06	Catalase-peroxidase 2

Table S4. Up-regulated functional genes in *M. oryzae* exposed to surfactin.

Gene ID	FoldChange (Sur vs CK)	log_2FoldChang e	*P*-value	Description
MGG_01353	1.935713453	0.952865404	0.000479977	Cytochrome P450 monooxygenase
MGG_00832	3.056353057	1.611811207	0.006118939	Cytochrome P450
MGG_09920	1.897171373	0.923850004	0.012057739	Cytochrome P450 52A5
MGG_00572	1.73350452	0.793691598	0.019040048	Cytochrome P450
MGG_08494	1.803209465	0.850566993	0.023104866	Cytochrome P450 52A13
MGG_04349	2.013676044	1.009831604	0.211909004	Cytochrome P450
MGG_04628	5.759087848	2.525840329	0.243024559	Cytochrome P450 51
MGG_07117	1.241890371	0.312537824	0.269089889	NADPH-cytochrome P450 reductase
MGG_07551	3.100886251	1.632680605	0.278526073	Cytochrome P450 monooxygenase
MGG_04911	1.222009983	0.289256071	0.404628633	Cytochrome P450 3A5
MGG_07626	1.147658798	0.198693789	0.442188311	Cytochrome P450 monooxygenase
MGG_01700	1.241088737	0.311606271	0.464587473	Cytochrome P450 monooxygenase
MGG_04469	1.26072427	0.334252781	0.544981249	Cytochrome P450 97B3
MGG_14591	4.823391004	2.270047766	0.676455518	Cytochrome P450 3A24

MGG_14217	1.503751979	0.588566636	0.818867758	Cytochrome P450 4F2 omega-hydroxylase
MGG_04304	46.88262723	5.550981513	1.3255E-06	Retinol dehydrogenase 8
MGG_10913	40.80639741	5.350723443	1.91575E-05	Retinol dehydrogenase 12
MGG_06534	14.56909804	3.864839659	3.8051E-05	Retinol dehydrogenase 12
MGG_01680	1.633947558	0.708361681	0.01709464	Retinol dehydrogenase 13
MGG_10613	5.457560877	2.448256318	0.021825669	Retinol dehydrogenase 11
MGG_07491	2.298843572	1.200908299	0.185998385	Retinol dehydrogenase 13
MGG_13624	4.080291602	2.028672259	0.000144014	ABC transporter CDR4
MGG_00416	3.858712803	1.948119671	0.757140609	MFS transporter
MGG_10710	3.912033078	1.967918569	0.000155675	Oxidoreductase
MGG_13440	1.009394402	0.013489991	0.986201853	Oxidoreductase
MGG_08349	28.36219955	4.825897516	0.0002243	Dehydrogenase/reductase SDR family member 13
MGG_00426	2.515285497	1.330722162	0.177589834	Short-chain dehydrogenase/reductase SDR
